# Transforming Abdominal Wall Surgery With Generative Artificial Intelligence

**DOI:** 10.3389/jaws.2023.12419

**Published:** 2023-11-27

**Authors:** Julio Mayol

**Affiliations:** Hospital Clinico San Carlos, Instituto de Investigación Sanitaria San Carlos, Universidad Complutense de Madrid, Madrid, Spain

**Keywords:** hernia, abdominal wall, surgery, AI, ChatGPT

## Introduction

Artificial intelligence (AI) is a transformative technology with profound implications for various domains of surgery [1], including surgical practice, research, education/training, and management. Generative AI (GAI) involves the use of algorithms and neural networks to generate new content, whether it’s images, text, or even surgical plans. In medicine, this technology can be a game-changer [2], offering innovative solutions to complex healthcare challenges. While it’s still a relatively nascent field, the applications of GAI are expanding rapidly, showing great promise for surgeons [3].

Large Language Models (LLMs) like GPT (Generative Pre-trained Transformer) are GAI solutions that utilize a deep learning architecture [4] known as “transformer.” The transformer architecture is particularly suited for processing sequences of data, making it apt for tasks involving natural language processing (NLP). In the GPT models, the transformer’s architecture is employed in a decoder-only variant. The model is trained on a large corpus of text data in an unsupervised manner, meaning it learns to predict subsequent words in a sequence by analyzing patterns in the data it was trained on. It’s this training process that makes GPT a “pre-trained” model. The architecture features multiple layers of self-attention mechanisms and feed-forward neural networks, which enable it to handle long-range dependencies in text and generate coherent and contextually relevant text over extended sequences. The GPT models, especially the later versions like GPT-3, are characterized by their large size, with GPT-3 having 175 billion parameters, which allows them to capture a vast amount of information and exhibit a remarkable understanding of natural language.

Value-based hernia surgery requires highly specialized training and personalized surgical approaches, which require advanced data analytics. GAI has the capacity to analyze vast datasets of patient information, historical surgical procedures, and outcomes to develop tailored surgical plans. Furthermore, during surgery, it can assist in real-time decision-making, providing surgeons with valuable insights that can enhance surgical precision. Additionally, in the context of education and training, generative AI-driven simulations can offer aspiring surgeons a realistic platform to practice and refine their skills before entering the operating room. Moreover, it holds great potential in optimizing the management of hernia patients, from preoperative planning to postoperative care. In summary, GAI stands as a transformative tool that has the potential to revolutionize hernia surgery in multiple dimensions [5], offering hope for improved outcomes and patient care. Some of the key elements of the use of GAI in hernia surgery will be discussed.

## Abdominal Wall Surgery and Generative AI: Practice, Training, and Research

GAI is not designed to replace the skill and expertise of experienced surgeons but rather to complement their abilities and enhance the quality of care provided to patients undergoing hernia surgery (Table 1). In the realm of surgical practice, generative AI can serve as a valuable assistant to seasoned surgeons, offering a wealth of benefits that can lead to improved patient outcomes.

**TABLE 1:** Generative AI solutions: potential applications to hernia surgery, and associated risks.

Generative AI solution	Potential application to hernia surgery	Associated risks
Image synthesis	Generation of anatomical structures to simulate various hernia cases for training	Inaccurate representation might lead to misunderstanding or misinterpretation of real-life cases
Data augmentation	Enhance the dataset of hernia images, making machine learning models more robust	Over-reliance on augmented data might introduce biases or errors
Patient-specific simulation	Personalized surgical plans and simulations based on individual patient data	If not accurate, may lead to suboptimal surgical decisions
Automated report generation	Producing post-operative reports based on surgical and patient data	Generated reports might miss critical details or contain inaccuracies
Predictive analysis	Predict potential complications or outcomes post hernia surgery based on generated data patterns	False predictions might lead to wrong clinical decisions or give false assurance
Virtual surgical assistants	Generative models to suggest surgical steps or provide real-time feedback	Over-reliance might reduce surgeon’s intuition or critical thinking during surgery

One of the primary ways in which generative AI aids expert surgeons is through surgical planning. Hernia surgery is inherently complex, with each case presenting unique challenges. GAI can analyze a patient’s medical history, imaging data, and other relevant information to create personalized surgical plans. By synthesizing these data, AI can provide surgeons with invaluable insights, helping them make informed decisions about the surgical approach, technique, and potential complications. These AI-generated plans act as a blueprint that can significantly enhance surgical precision.

Moreover, GAI’s potential shines brightest in real-time decision-making during surgery [6]. Even the most experienced surgeons can encounter unexpected situations in the operating room. AI systems, powered by machine learning algorithms, can process vast amounts of data in real-time, offering suggestions and predictions [7] to the surgeon, even based on the analysis of surgical video [8]. For example, during a hernia repair procedure, if unexpected tissue characteristics are encountered, AI can suggest alternative approaches or anticipate potential complications. This dynamic assistance can be a game-changer in maintaining patient safety and optimizing surgical outcomes.

GAI offers a remarkable opportunity to advance the fields of surgical training and research, revolutionizing the way future surgeons are educated [9] and how medical insights are gained through the analysis of vast datasets. This transformative technology has the potential to bridge the gap between traditional medical education and the demands of modern healthcare.

For surgical training, GAI can play a pivotal role in preparing the next-generation of surgeons. AI-driven simulations are particularly valuable in this regard [10]. These simulations, powered by GAI algorithms, provide aspiring surgeons with a highly realistic and interactive environment to practice surgical techniques, hone their skills, and familiarize themselves with complex procedures like hernia repair. Through AI simulations, trainees can experience a wide range of surgical scenarios, from routine cases to challenging complications, without any risk to real patients and with highly reliable immediate feedback [11]. This not only accelerates their learning curve but also instills a sense of confidence and competence that is crucial for success in the operating room.

Furthermore, AI technology contributes significantly to surgical research [6] by enabling the analysis of vast and complex medical datasets. In the context of hernia surgery, AI can extract valuable insights from electronic health records, medical imaging, and patient outcomes data. By identifying patterns, predicting patient responses, and suggesting potential areas of improvement, GAI can guide researchers in developing more effective surgical techniques and treatment strategies. This data-driven approach has the potential to accelerate the pace of medical discovery and enhance our understanding of hernia, ultimately leading to improved patient care and outcomes.

## Empowering Patients

GAI is not only a tool for surgeons but also a means to empower patients [12] with comprehensive information and support throughout their hernia surgery journey. By leveraging AI-driven applications, patients can gain a better understanding of their condition, surgical procedure, and postoperative care, in a more empathic manner [13] ultimately leading to improved patient engagement and outcomes.

One of the key ways LLMs empower patients is through education about their condition. Hernia surgery can be a daunting prospect for many patients, often shrouded in medical jargon and complexity. GAI-powered educational tools can bridge this knowledge gap by providing accessible, personalized information [14]. For instance, generative AI can generate easy-to-understand visualizations or videos that explain the specific type of hernia a patient has, the anatomy involved, and the steps of the surgical procedure. This empowers patients to make informed decisions about their treatment and alleviates anxiety through enhanced clarity.

**FIGURE 1:**
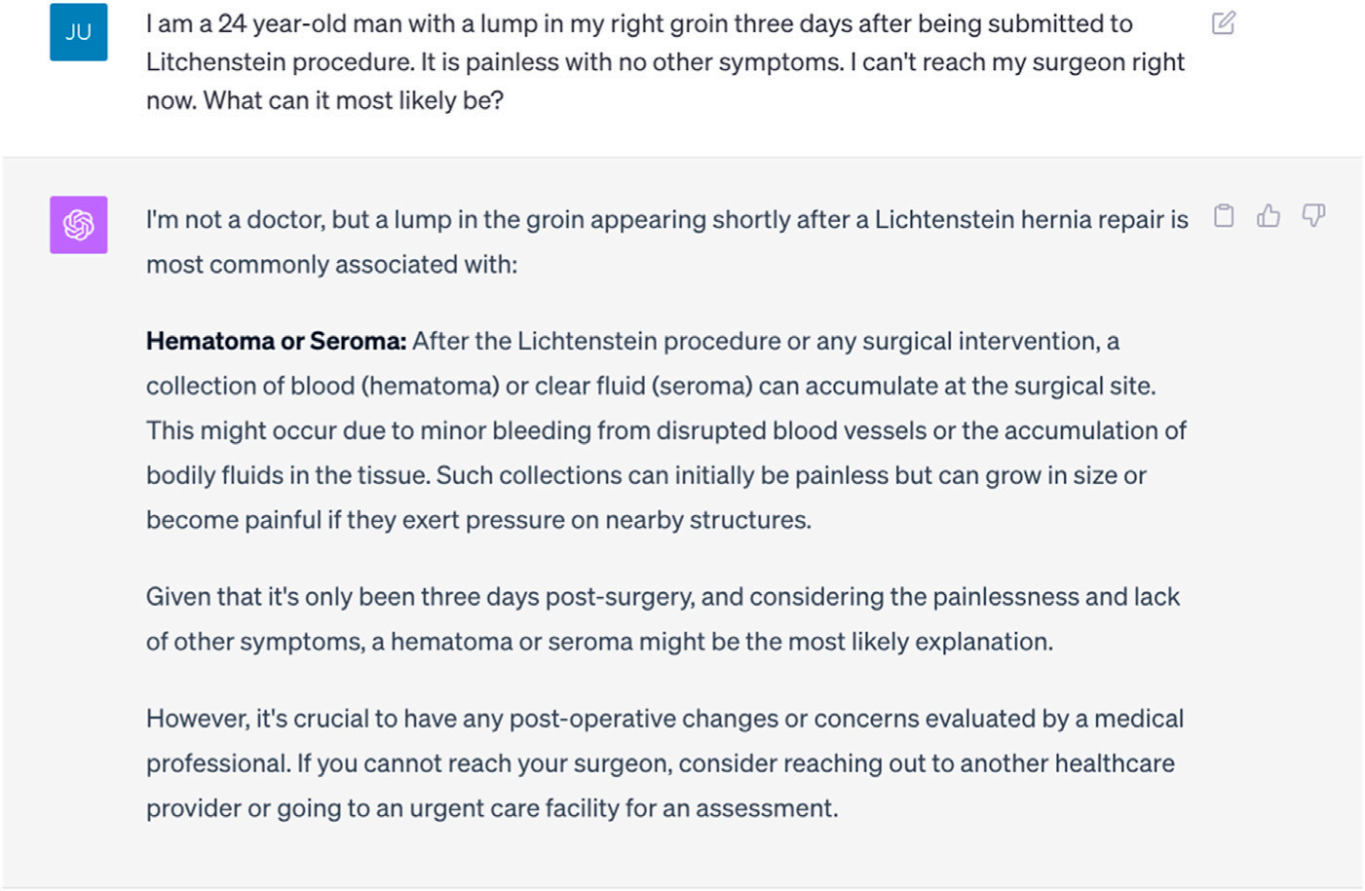
A ChatGPT (GPT4) response to a simulated question related to a postoperative complication.

Additionally, GAI applications can play a crucial role in enhancing postoperative recovery and follow-up care [15]. Chatbots can provide patients with personalized recovery plans, offering guidance on post-surgery exercises, diet, and medication schedules. As shown in Figure 1, ChatGPT can answer patients’ questions about potential postoperative complications. These AI-driven applications can monitor patients’ progress, collecting data on vital signs and symptoms, and automatically alert healthcare providers if any concerning trends are detected. By facilitating remote monitoring and timely interventions, AI promotes a smoother recovery process and reduces the need for frequent in-person appointments, which is especially valuable in today’s digital healthcare landscape.

## Discussion

The integration of GAI into hernia surgery, while promising, brings forth a set of challenges and ethical considerations that must be carefully addressed to ensure patient safety, privacy, and ethical practice as in any other healthcare domain [16, 17].

One of the foremost concerns involves the ethical implications of AI in medicine. As AI systems become increasingly sophisticated, the question arises: to what extent should AI be involved in decision-making during hernia surgery? Striking the right balance between AI assistance and human expertise is critical. Surgeons must remain the ultimate decision-makers, and AI should be viewed as a valuable tool that offers insights and recommendations. The ethical challenge lies in defining the boundaries of AI’s role and ensuring that it does not compromise patient safety or the surgeon’s professional judgment [18].

Patient data privacy is another ethical consideration. AI systems in hernia surgery rely on vast datasets that often contain sensitive patient information. Ensuring the security and confidentiality of this data is paramount. Hospitals and healthcare institutions must implement robust data protection measures, including encryption and access controls, to safeguard patient privacy. Additionally, patients should be fully informed about how their data will be used and have the option to opt out if they have concerns. Transparency in data handling is crucial to maintain trust between patients, healthcare providers, and AI systems [19].

Proper supervision and regulation are essential to mitigate ethical challenges and ensure the responsible use of AI in hernia surgery. Regulatory bodies should establish guidelines and standards for the development and deployment of AI systems in healthcare. These regulations should encompass AI safety, data security, accountability, and transparency. Surgeons and healthcare providers must also undergo comprehensive training to understand the capabilities and limitations of AI systems and to use them ethically and responsibly.

Finally, here are the ten recommendations for hernia surgeons who want to make the most out of GAI for value-based surgery once the technology approved:

1. Personalized surgical plans for better outcomes: utilize GAI to develop personalized surgical plans based on individual patient data to enhance the quality and effectiveness of care, thereby improving surgical outcomes and patient satisfaction.
2. Efficiency and cost-effectiveness: incorporate GAI to streamline surgical procedures, reducing operative time, and resources utilized, which in turn could lead to cost savings and enhanced value.
3. Preoperative patient education for informed decisions: leverage AI-driven tools for educating patients about their hernia condition and the surgical procedure, helping them make informed decisions and setting realistic expectations.
4. Postoperative monitoring for improved recovery: use AI-powered applications for real-time postoperative monitoring to accelerate recovery, reduce readmissions, and minimize post-surgical complications, thereby enhancing value.
5. Telehealth consultations for continuous care: employ AI-powered telehealth solutions for postoperative consultations, ensuring continuous care while reducing the necessity of in-person visits and associated costs.
6. Data-driven research for innovation: leverage GAI to analyze extensive medical datasets to drive research and innovation in hernia surgery, developing new, cost-effective treatment strategies.
7. Efficient clinical trials: utilize GAI to expedite the conduct of large-scale clinical trials, accelerating the evaluation and adoption of innovative, value-enhancing surgical techniques and medical devices.
8. Continuous learning: stay updated with the latest advancements in GAI technology and engage in continuous learning to improve the quality of hernia surgical care and patient outcomes.
9. Multidisciplinary collaboration: foster multidisciplinary collaboration with AI experts and other medical specialties to develop holistic, value-based GAI solutions for hernia surgery.
10. Patient-centered care: embrace a patient-centered approach, leveraging GAI to enhance the overall patient care journey, aiming for improved long-term outcomes, patient satisfaction, and value.

In conclusion, AI holds immense promise in the field of hernia surgery. It envisions a healthcare landscape where each patient receives personalized, data-driven care, and where GAI supports the entire patient journey. Moreover, GAI’s potential to advance research is poised to deepen our understanding of these conditions to deliver better value to our patients.
